# Supplementary material for: Both genome instability and replicative senescence stem from the shortest telomere in telomerase-negative cells
Source: Nat Commun. 2026 Mar 15;17:4271. doi: 10.1038/s41467-026-70352-z (PMC13168691; doi:10.1038/s41467-026-70352-z)
Supplement: Supplementary file 1 — Supplementary Information [file 41467_2026_70352_MOESM1_ESM.pdf]

Supplemental Information

# Both Genome Instability and Replicative Senescence Stem from the Shortest Telomere in Telomerase-Negative Cells

Prisca Berardi[1,#]. Veronica Martinez-Fernandez[1,#]. Anaïs Rat[2]. Fernando R. Rosas Bringas[3]. Pascale Jolivet[1]. Rachel Langston[1]. Stefano Mattarocci[1]. Alexandre Maes[1]. Théo Aspert[4,5,6,7]. Bechara Zeinoun[1]. Karine Casier[1]. Hinke G. Kazemier[3]. Gilles Charvin[4,5,6,7]. Marie Doumic[8]. Michael Chang[3] and Maria Teresa Teixeira[1,*]

Author affiliations

[1] Sorbonne Université. CNRS. Laboratoire de Biologie Moléculaire et Cellulaire des Eucaryotes. LBMCE. F-75005 Paris. France
[2] Univ Brest. CNRS. UMR 6205. Laboratoire de Mathématiques de Bretagne Atlantique. 29200 Brest. France
[3] European Research Institute for the Biology of Ageing. University of Groningen. University Medical Center Groningen. Groningen. the Netherlands
[4] Department of Developmental Biology and Stem Cells. Institut de Génétique et de Biologie Moléculaire et Cellulaire. Illkirch. France.
[5] CNRS. UMR7104. Illkirch. France.
[6] INSERM. U964. Illkirch. France.
[7] Université de Strasbourg. Illkirch. France.
[8] CMAP. Inria. IP Paris. Ecole polytechnique. CNRS. 91128 Palaiseau cedex. France.
[#] The authors contributed equally (by alphabetical order)
*Correspondence: teresa.teixeira@cnrs.fr

Contents:

Supplementary Fig. 1-7
Supplementary Tables 1-13
Supplementary Pipeline 1
Supplementary Pipeline 2
References

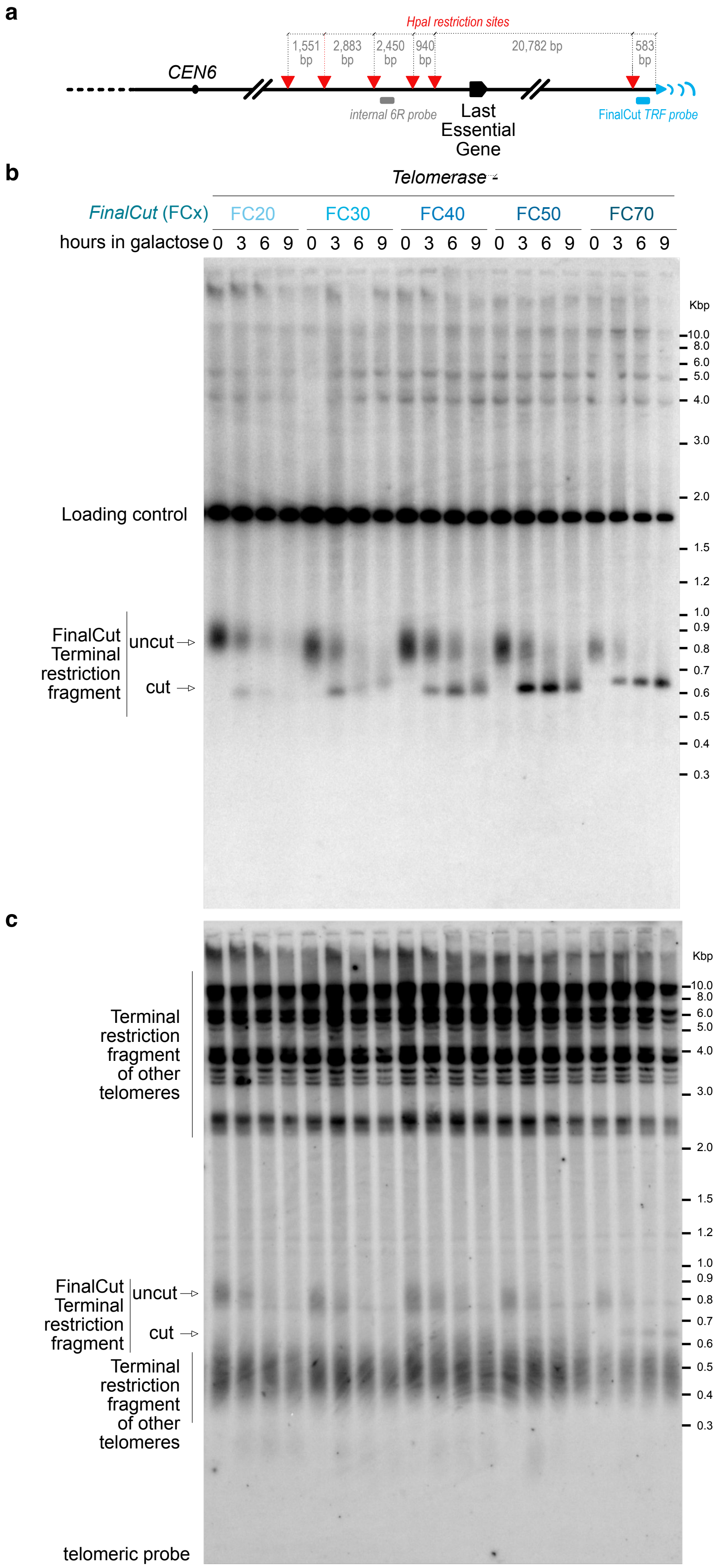
a
HpaI restriction sites
1,551 bp
2,883 bp
2,450 bp
940 bp
20,782 bp
583 bp
CEN6
internal 6R probe
Last Essential Gene
FinalCut TRF probe
b
Telomerase
FinalCut (FCx)
FC20
FC30
FC40
FC50
FC70
hours in galactose
0 3 6 9 0 3 6 9 0 3 6 9 0 3 6 9 0 3 6 9
Kbp
10.0
8.0
6.0
5.0
4.0
3.0
2.0
1.5
1.2
1.0
0.9
0.8
0.7
0.6
0.5
0.4
0.3
Loading control
FinalCut Terminal restriction fragment
uncut
cut
c
Kbp
Terminal restriction fragment of other telomeres
FinalCut Terminal restriction fragment
uncut
cut
Terminal restriction fragment of other telomeres
telomeric probe

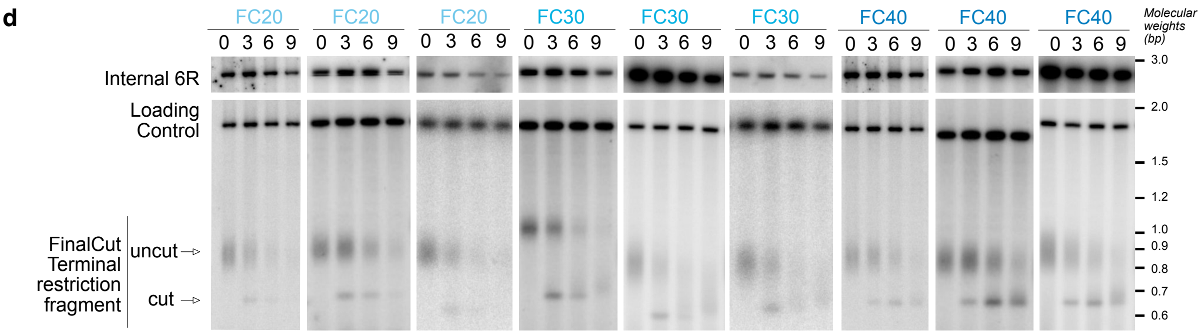

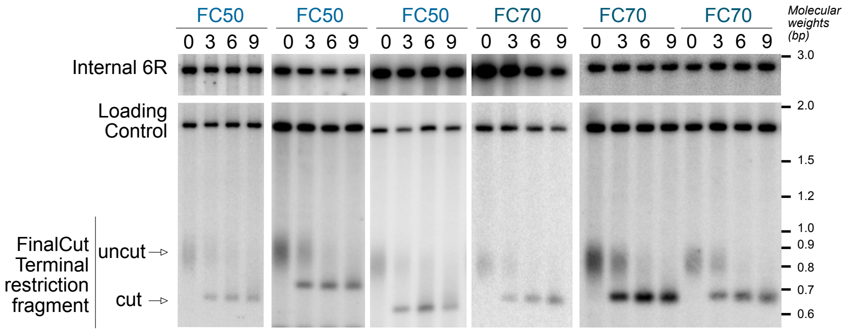

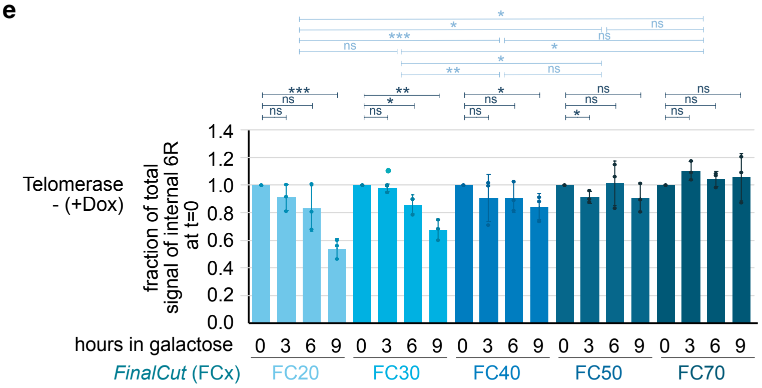

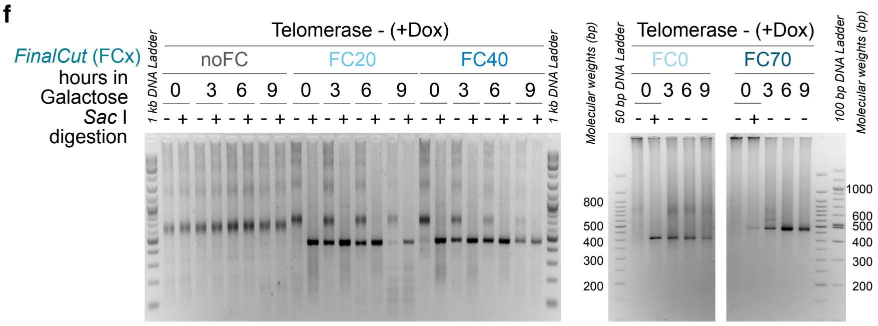

Supplementary Fig. 1: Related to Fig.1.

a, *Restriction map of the end of chromosome 6R arm (not to scale).*

b-c, *The Southern blot shown in Figure 1d shown in full (b) was rehybridized with an oligo probe targeting telomeric repeats (c). Source data are provided as a Source Data file.*

d, *Triplicate individual Southern blots of TRF of* Hpa I *digested genomic DNA, prepared at indicated time points from strains with indicated* FinalCut *telomere lengths in the absence of telomerase, probed with a subtelomeric 6R to detect* FinalCut*, an internal locus in chromosome 11 (Loading control), and an internal region of chromosome 6 R arm 23 kb away from* FinalCut*, used for quantifications shown in Supplementary Fig. 1e. Molecular weights are indicative for one of the blots (different migrations/different blots may apply). Source data are provided as a Source Data file.*

e, *Stability of the right arm of chromosome 6 at 23 kb from the FC telomere as quantified from Southern Blots shown in d. N=3 independent experiments. Error bars correspond to SD. Statistical differences between 0h and remaining time points of "uncut" were determined by two-sided Student t-tests (dark blue lines). Differences in the decay kinetics of the "internal 6R" among the* FinalCut *constructs were evaluated by comparing the slopes of linear regressions using a one-way ANOVA (p-value=0.0012, not depicted). Differences in the stability of the "internal 6R" over time among the* FinalCut *constructs were evaluated by comparing the slopes of linear regressions with two-sided Student's t-tests (light blue lines). *: $p < 0.05$; **: $p < 0.005$; ***: $p < 0.0005$; ns: not significant. Source data are provided as a Source Data file.*

f, *Efficiency of Cas9-dependent* FinalCut *cleavage was also qualitatively verified by TeloPCR of* TEL6R*. Here, representative result of* TEL6R*-specific telo-PCR with genomic DNA of indicated strains incubated in doxycycline and galactose for indicated hours. If indicated, PCR product was digested with* Sac I *restriction enzyme at 37°C for 1 hour. Sac I cleaves intact ttDNA (see Fig. 1a). TeloPCR products were migrated in 2.5% agarose gels.* TEL6R*-specific primer oT883 binds 6R subtelomere at 370 bp from the sub-telomere-telomere junction. Source data are provided as a Source Data file.*

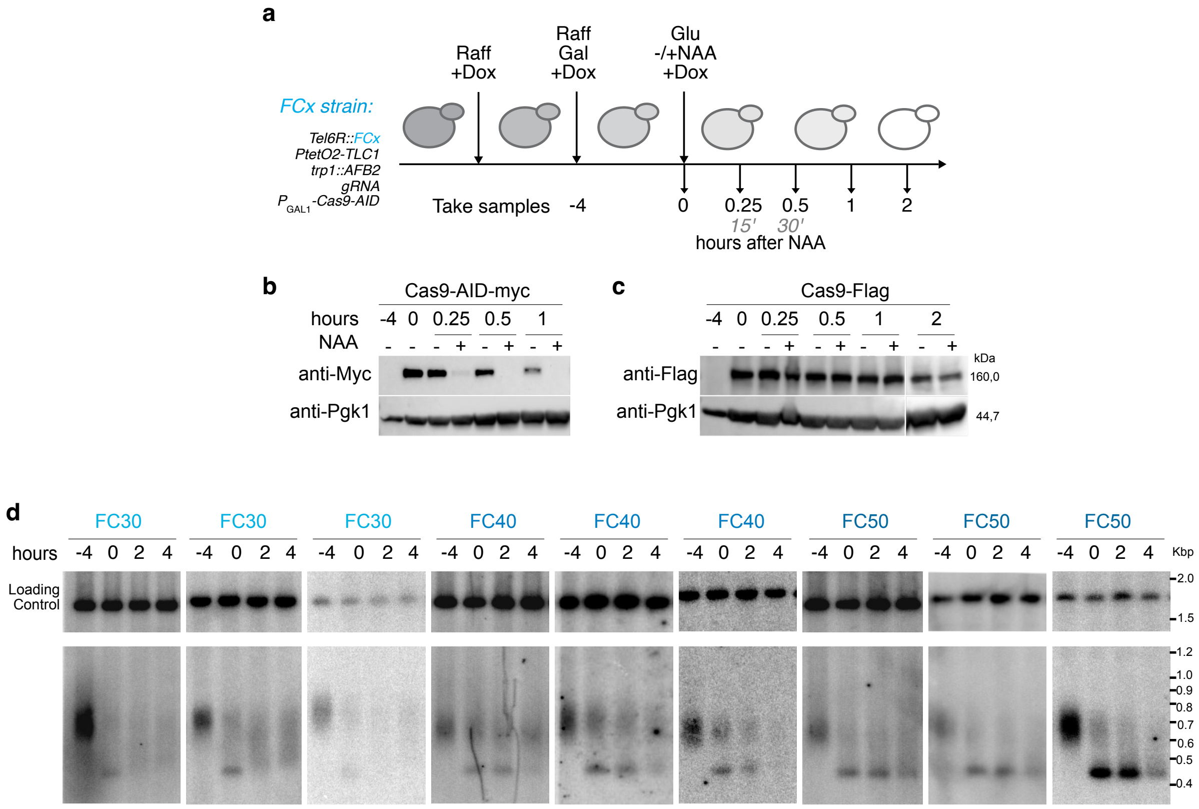

Supplementary Fig. 2: Related to Fig. 2.

a, Experimental design for testing Cas9-AID degradation.

***b-c,** Representative western blot of Cas9-AID fused to a Myc-tag (b) or a Cas9 without AID fused to a Flag-tag (c) in a FC70 strain. An anti-Pgk1 serves as loading control. Source data are provided as a Source Data file.*

***d,** Individual Southern blots of TRF of* Hpa *I digested genomic DNA, prepared at indicated time points from strains with indicated* FinalCut *telomere lengths in the absence of telomerase, probed with the subtelomere 6R and an internal locus in chromosome 11 (Loading control), used for quantifications shown in Fig. 2c. Source data are provided as a Source Data file.*

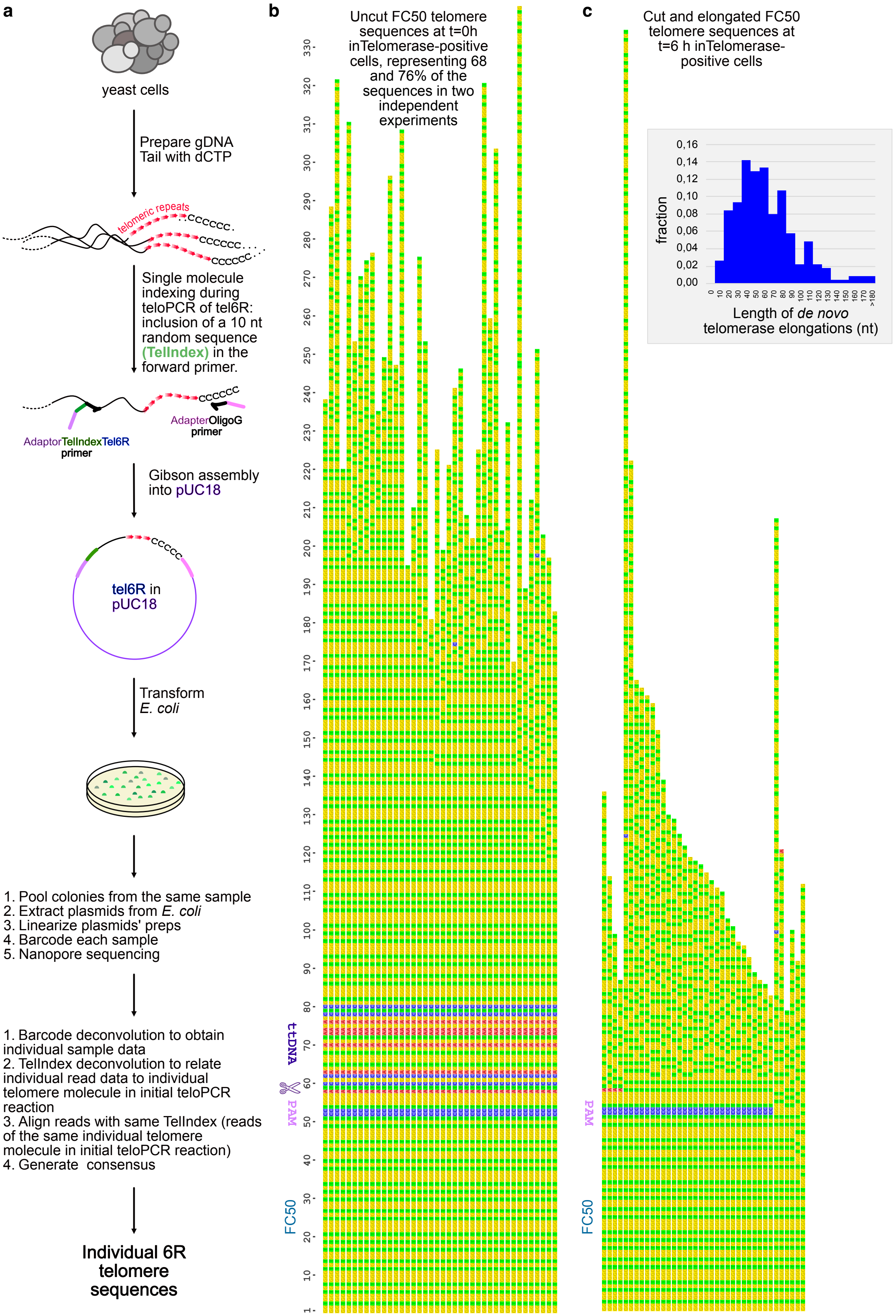
a
yeast cells
Prepare gDNA
Tail with dCTP
telomeric repeats
CCCCCC
Single molecule indexing during teloPCR of tel6R: inclusion of a 10 nt random sequence (TelIndex) in the forward primer.
AdaptorTelIndexTel6R primer
AdapterOligoG primer
Gibson assembly into pUC18
tel6R in pUC18
Transform E. coli
1. Pool colonies from the same sample
2. Extract plasmids from E. coli
3. Linearize plasmids' preps
4. Barcode each sample
5. Nanopore sequencing
1. Barcode deconvolution to obtain individual sample data
2. TelIndex deconvolution to relate individual read data to individual telomere molecule in initial teloPCR reaction
3. Align reads with same TelIndex (reads of the same individual telomere molecule in initial teloPCR reaction)
4. Generate consensus
Individual 6R telomere sequences
b
Uncut FC50 telomere sequences at t=0h inTelomerase-positive cells, representing 68 and 76% of the sequences in two independent experiments
t-tDNA
PAM
FC50
c
Cut and elongated FC50 telomere sequences at t=6 h inTelomerase-positive cells
fraction
Length of de novo telomerase elongations (nt)
PAM
FC50

d

FC50 telomere sequences at t=0 h inTelomerase-negative cells

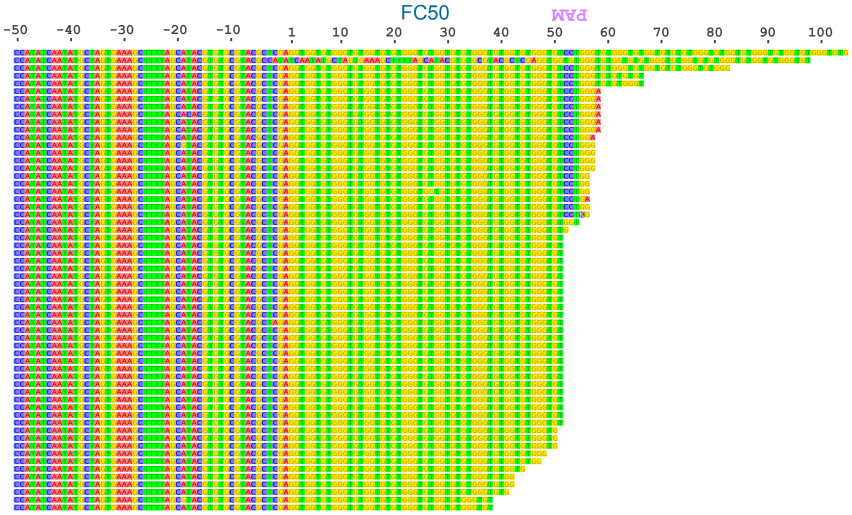

e

FC50 telomere sequences at t=2 h inTelomerase-negative cells

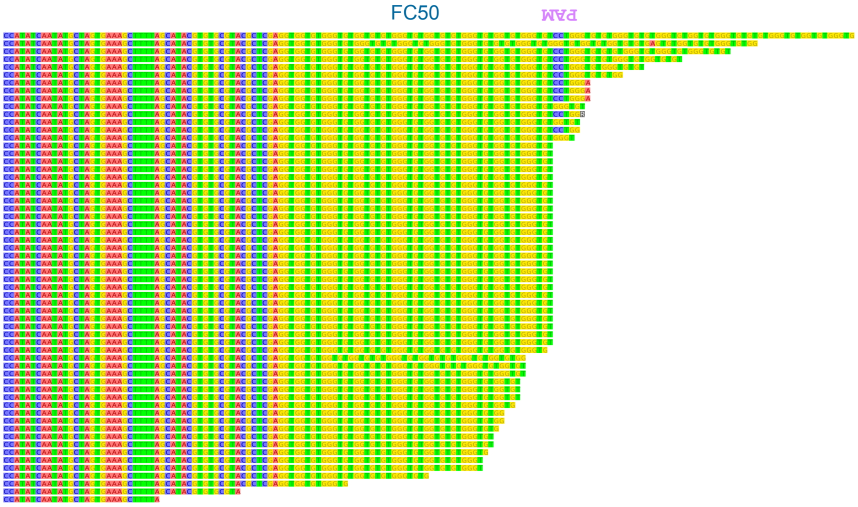

f

FC50 telomere sequences at t=4 h inTelomerase-negative cells

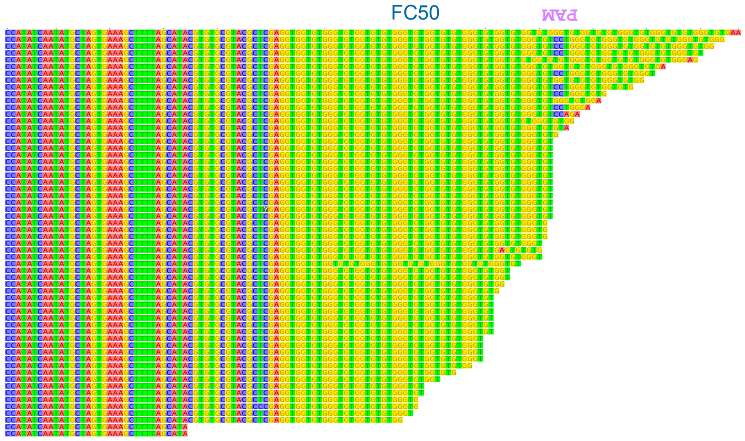

g

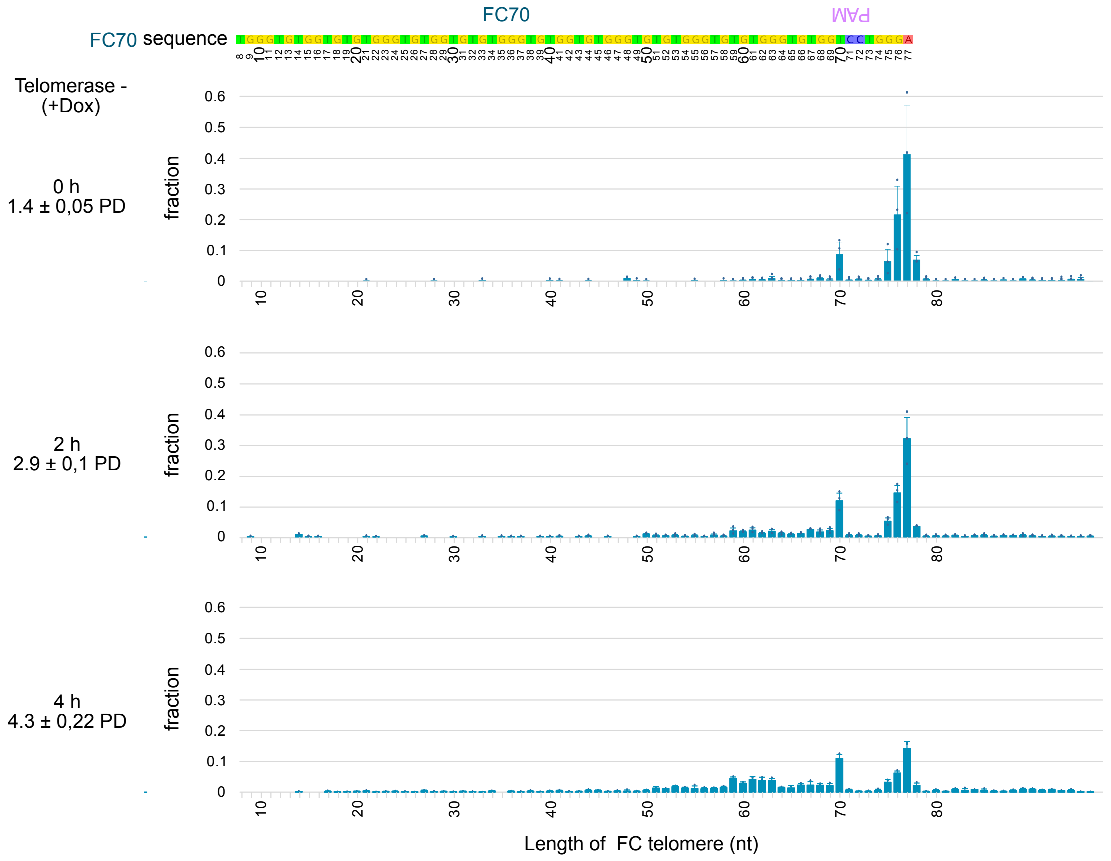

Supplementary Fig. 3: Related to Fig. 3.

a, *Protocol of sample preparation to sequence of hundreds of individual telomeres with high accuracy.*

b-f, *Examples of sequences of individual* FC50 *telomeres before (b) or after indicated times in Galactose induction of Cas9 during 4 h and subsequent shift into glucose and NAA in the absence (c) or presence of doxycycline (d-e). 20 consensus sequences of 2 (b), 19 of 2 (c) or 20 of 3 (d-e) independent experiments are shown for each time point. Sequences were edited for single mismatches sequencing errors. Inset in (c) corresponds to the distribution of telomerase-dependent re-elongations evaluated from sequence divergence.*

g, *Distribution of individual telomere lengths with time after Cas9 cut as in Fig.3f using FC70 strain in the absence of telomerase (+Dox) for indicated range of telomere lengths/Magnified view of Fig.3e. Dots represent the average of each of 3 independent experiment in which N=1119, 1109, 764 for t=0 h; N=819, 831, 1416 for t=2 h; N=1102, 1018, 745 for t=4 h individual telomere sequences, as indicated in the legend of Fig. 3e. Error bars correspond to SD from the average of 3 independent experiments. Source data are provided as a Source Data file.*

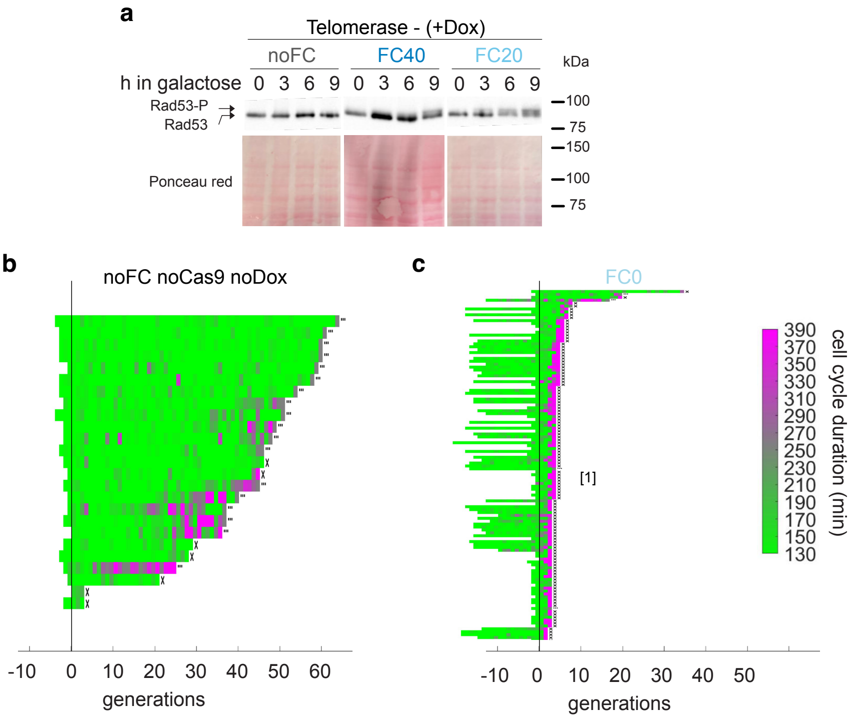

Supplementary Fig. 4: Related to Fig. 4.

a, *Indicated* FinalCut *strains were cultured in media containing doxycyclin and carbon source was changed to galactose for indicated time. Proteins were extracted and Rad53 phosphorylation was monitored by Western blot using an anti-Rad53 antibody. Ponceau red staining is shown as loading control. Source data are provided as a Source Data file.*

b-c, *Microfluidics positive and negative controls. Display of consecutive cell cycle durations of lineages of indicated strains. Experimental scheme and legends as in Fig. 4c. For b, recording was stopped at 5 days post injection into the microfluidics circuit (note many lineages haven't died at the end of the experiment (labelled with "..."). Source data are provided as a Source Data file.*

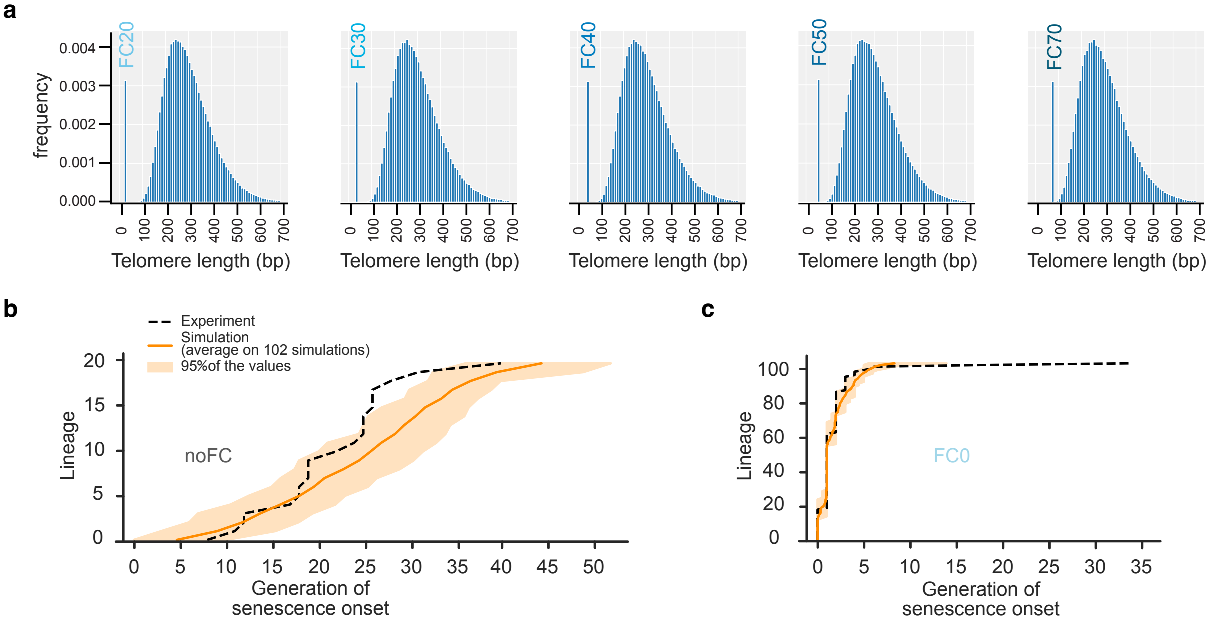

Supplementary Fig. 5: Related to Fig. 4.

a, *Telomere length distribution in cells that experienced a cut across 50 000 simulated lineages, with the mathematical model set for indicated* FinalCut *conditions. Values above 700 bp were truncated for clarity.*

b-c, *Lifespan of individual cell lineages entering senescence upon telomerase inactivation, as assessed by microfluidics in Supplementary Fig. 4b-c, and 102 simulations of the same number of individual cell lineages with the mathematical model set for indicated* FinalCut *conditions, ordered by lifespan.*

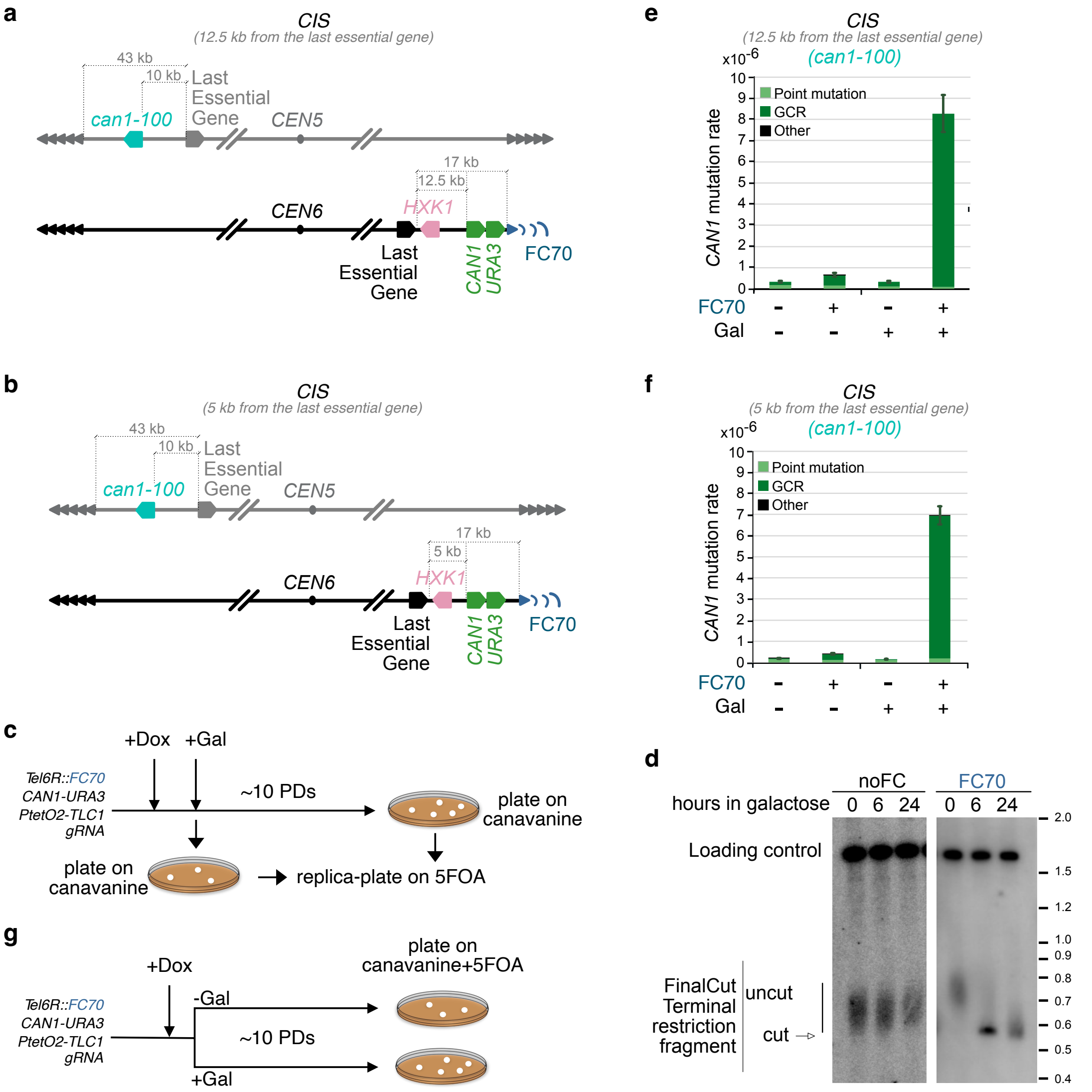

Supplementary Fig. 6: Related to Fig. 6.

a-b, *Relevant genomic features of strains used to quantify genomic instability at 12.5 kb (a: yT1696) or 5 kb (b: yT1698) from the last essential gene and in cis of* FC70 *. Legends as in Fig. 6a-b.*

c, *Experimental protocol for the fluctuation assay in cells harboring a* FC70 *telomere to determine CAN1 mutation rate. Multiple cultures are grown in parallel in which telomerase is inactivated by doxycycline addition, followed by carbon source switch to galactose to induce* FC70*. Then, a portion of each culture is plated at t=0 h and t=24 h after Cas9 induction on canavanine-containing glucose media lacking doxycycline. This allows for Cas9 shut off, telomerase re-expression to stabilize the genome and selection of mutants at* CAN1 *locus. Can^R colonies number distribution is used to estimate mutation rate according to fluctuation assay mathematical models. Can^R cells are then replica-plated on plates containing 5-FOA to score the fraction of GCRs or point mutations.*

d, *Southern blot of strains carrying indicated constructs at* TEL6R *incubated for indicated hours in galactose with probe targeting the* FinalCut *TRF and loading control. Strains used: yT576 and yT1612.*

e-f, *Mutation rate of* CAN1 *gene, located either 12.5 kb (d) or 5 kb (e) from the last essential gene and in cis of* noFC *or* FC70 *in* can1-100 *cells, calculated from cells plated at 0 h and 24 h after Cas9 induction (see Supplementary Table 9). Data are represented as the mutation rate ±95% confidence interval. Mutation type distribution of Can^R colonies obtained at 0 h and 24 h after Cas9 induction is indicated. Others correspond to a very small fraction of colonies [Can^R 5-FOA^R ura$^-$]. See Supplementary Table 10 for statistical details. Strain used in d: yT1696* (FC70) *and yT1710* (noFC, "FC70 -")*. Strain used in e: yT1698* (FC70) *and yT1712* (noFC, "FC70 -")*. Source data are provided as a Source Data file.*

g, *Experimental protocol for the fluctuation assay to assess* CAN1-URA3 *mutation rate (GCRs). Multiple cultures are grown in parallel in which telomerase is inactivated by doxycycline addition, followed by carbon source switch to galactose to induce* FC70 or not. *Upon ~10 PDs, the cultures are plated on canavanine and 5-FOA-containing glucose media lacking doxycycline. This allows for Cas9 shut off, telomerase re-expression to stabilize the genome and selection of mutants at both* CAN1 *and* URA3 *locus. Can^R 5FOA^R ura3$^-$ colonies number distribution is used to estimate GCR rate according to fluctuation assay mathematical models.*

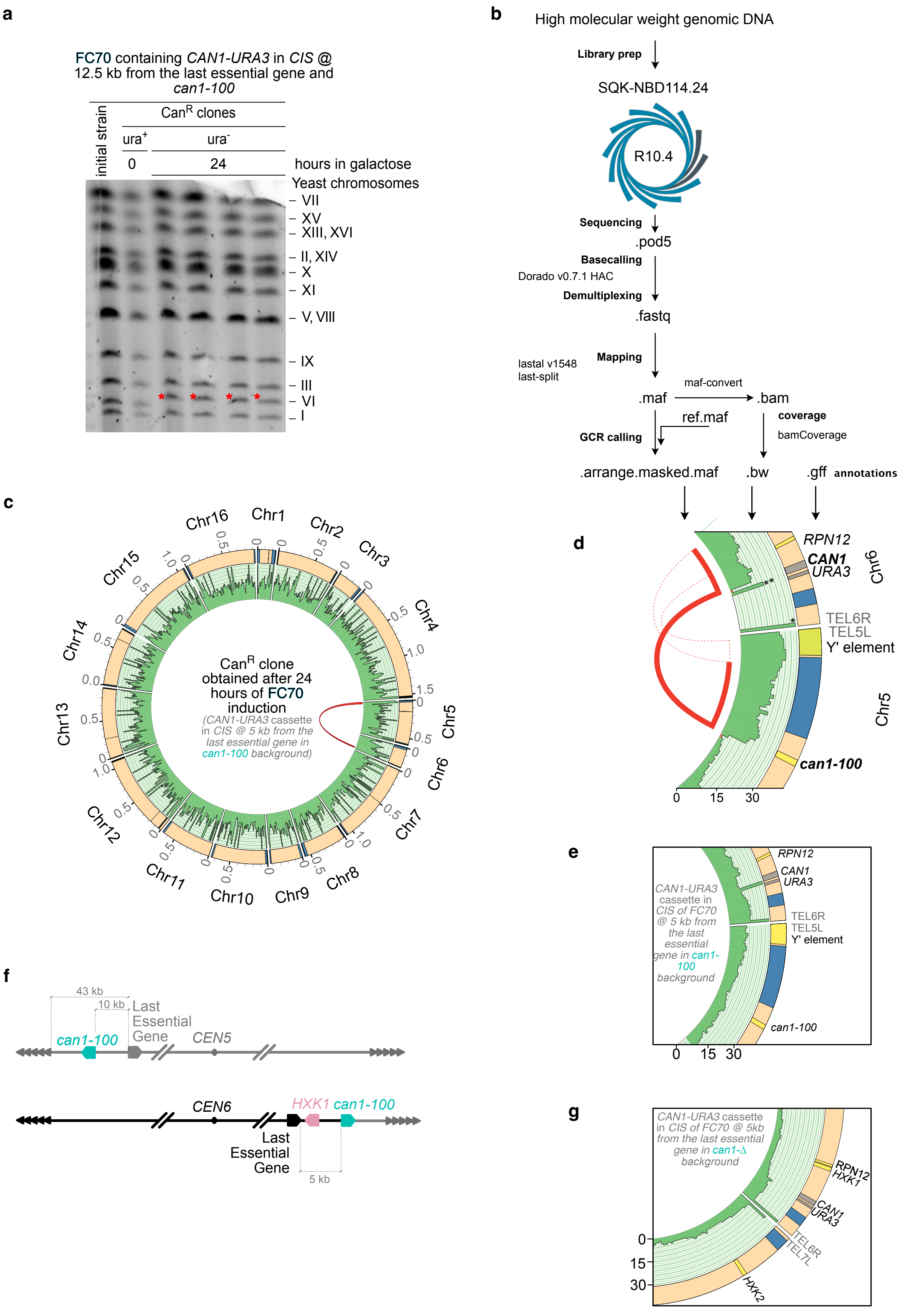
a
FC70 containing CAN1-URA3 in CIS @ 12.5 kb from the last essential gene and can1-100
initial strain
CanR clones
ura+
ura-
0
24
hours in galactose
Yeast chromosomes
VII
XV
XIII, XVI
II, XIV
X
XI
V, VIII
IX
III
VI
I
b
High molecular weight genomic DNA
Library prep
SQK-NBD114.24
R10.4
Sequencing
.pod5
Basecalling
Dorado v0.7.1 HAC
Demultiplexing
.fastq
Mapping
lastal v1548
last-split
maf-convert
.maf
.bam
ref.maf
coverage
bamCoverage
GCR calling
.arrange.masked.maf
.bw
.gff
annotations
c
CanR clone obtained after 24 hours of FC70 induction
(CAN1-URA3 cassette in CIS @ 5 kb from the last essential gene in can1-100 background)
d
RPN12
CAN1
URA3
Chr6
TEL6R
TEL5L
Y' element
Chr5
can1-100
e
CAN1-URA3 cassette in CIS of FC70 @ 5 kb from the last essential gene in can1-100 background
f
43 kb
10 kb
Last Essential Gene
can1-100
CEN5
CEN6
HXK1
can1-100
Last Essential Gene
5 kb
g
CAN1-URA3 cassette in CIS of FC70 @ 5kb from the last essential gene in can1-Δ background
RPN12
HXK1
CAN1
URA3
TEL6R
TEL7L
HXK2

h

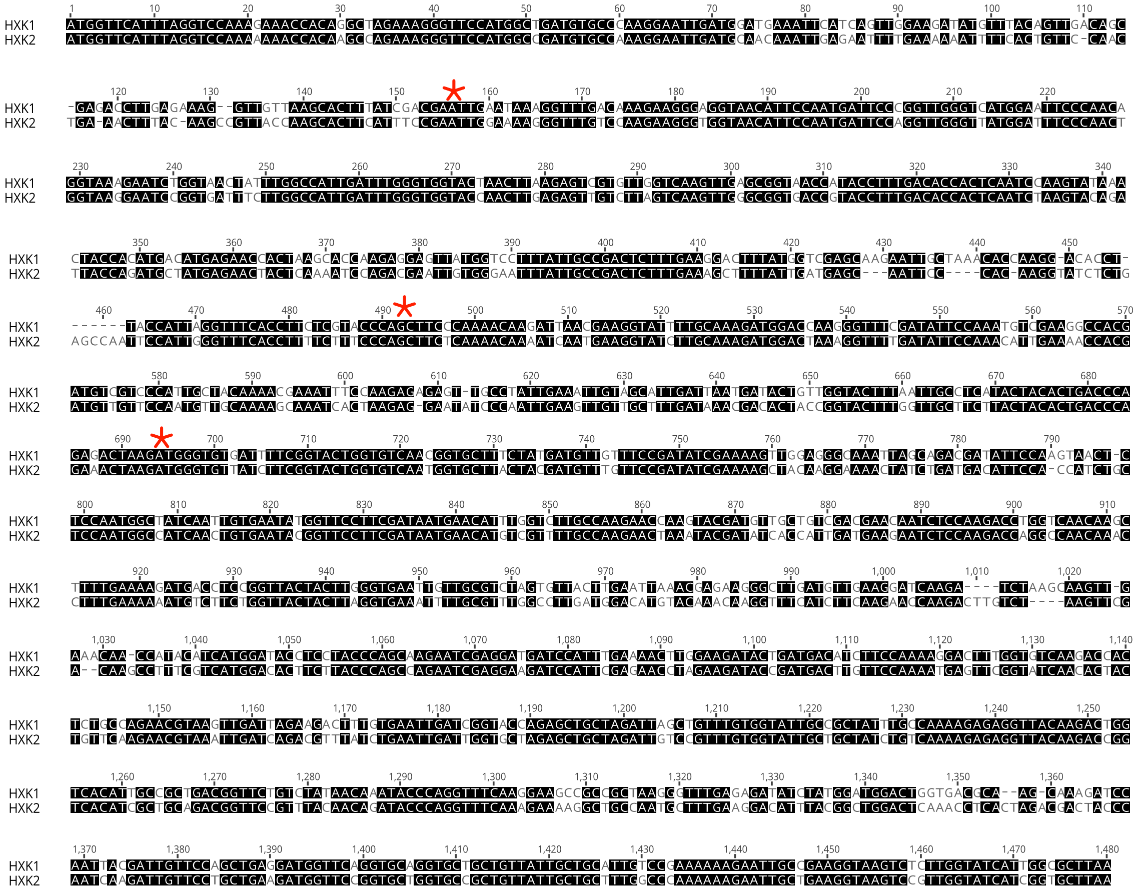

Supplementary Fig. 7 (Related to Figure 7). Genomic instability at the shortest telomere in the cell enables senescence escape.

a, *Pulse-Field Gel Electrophoresis of indicated strains (derived from initial strain yT1696, grown for indicated time in galactose. Red asterisk indicates a change in chromosome 6 mobility. Chromosomes 4 and 12 run upper and were excluded from the picture.*

b, *Pipeline for generating the CIRCOS based genome assembly.*

c, *CIRCOS plot showing the genome of a Can^R 5-FOA^R ura$^-$ colony classified in this study as GCR, obtained after 24 h of Cas9 induction in the absence of telomerase of cells harboring a FC70 telomere, the CAN1-URA3 cassette at 5 kb from the last essential gene on chromosome 6R and in a can1-100 backgound (initial strain: yT1698, Supplementary Fig. 6b-c, f). Legends as in Fig. 7a.*

d, *CIRCOS plot detail of c showing* loci *involved in the breakpoint region, including junction* CAN1-can1-100 *in bold. Chromosome 5 and 6 were tilted to achieve juxtapositition of* TEL5L *and* TEL6R *in the scheme. The solid red lines represents the longest splitted read. Dashed red lines indicate the extremities of splitted reads. (*) Reads containing exclusively TG$_{1-3}$, which map telomeric regions indiscriminately. (**) Reads mapping a region of the* CAN1-URA3 *cassette (in grey) containing a short plasmid-derived sequence (60 bp), also present at the* AFB2-TRP1 *locus.*

e, *CIRCOS plot of the genome of control strain yT1698 zoomed into relevant region implicated in the rearangement showed in d.*

f, *proposed model for the chromosomal rearrangement involving chromosomes 6 and 5 of Can^R.*

g, *CIRCOS plot of genomes of control strain yT1760 zoomed into relevant regions implicated in the rearangement showed Fig. 7.*

h, *Alignment of HXK1 and HXK2 present in yT1760. Homologous sequences are highlighted in black. Recombination regions characterized by ONT from three independent Can^R mutants are indicated with red stars.*

Supplementary Table 1: Yeast strains used in this work

Name	Comment	Figure	Genotype
G49	Obtained from M. Lisby		*MATα ura3-1 leu2-3.112 his3-11.15 can1-100*
yT992			*MATα ura3-1 leu2-3.112 his3-11.15 can1-100 tlc1::HIS3MX6-*P_{Teto2}*-TLC1*
yT1487	yT992 transformed with Eco RV-digested YIp204-PADH1-AFB2	S1.S4	*MATα ura3-1 trp1-1::AFB2-TRP1 leu2-3.112 his3-11.15 can1-100 tlc1::HIS3MX6-*P_{Teto2}*-TLC1*
yT1529	yT1487/pT182	S1.S4	*MATα ura3-1 trp1-1::AFB2-TRP1 leu2-3.112 his3-11.15 can1-100 tlc1::HIS3MX6-*P_{Teto2}*-TLC1 TEL6R::FC0*
yT1503	yT1487/pT177	1.4.S1	*MATα ura3-1 trp1-1::AFB2-TRP1 leu2-3.112 his3-11.15 can1-100 tlc1::HIS3MX6-*P_{Teto2}*-TLC1 TEL6R::FC20*
yT1504	yT1487/pT176	1-2.4.S1	*MATα ura3-1 trp1-1::AFB2-TRP1 leu2-3.112 his3-11.15 can1-100 tlc1::HIS3MX6-*P_{Teto2}*-TLC1 TEL6R::FC30*
yT1505 yT1527	yT1487/pT175	1-2.4.S1	*MATα ura3-1 trp1-1::AFB2-TRP1 leu2-3.112 his3-11.15 can1-100 tlc1::HIS3MX6-*P_{Teto2}*-TLC1 TEL6R::FC40*
yT1506	yT1487/pT174	1-2.4.S1	*MATα ura3-1 trp1-1::AFB2-TRP1 leu2-3.112 his3-11.15 can1-100 tlc1::HIS3MX6-*P_{Teto2}*-TLC1 TEL6R::FC50*
yT1576	yT1507 backcrossed with G49	S6	*MATa ura3-1 trp1-1::AFB2-TRP1 leu2-3.112 his3-11.15 can1-100 tlc1::HIS3MX6-*P_{Teto2}*-TLC1*
yT1597	yT1487/pT187	1-4.S1-3	*MAT α ura3-1 trp1-1::AFB2-TRP1 leu2-3.112 his3-11.15 can1-100 tlc1::HIS3MX6-*P_{Teto2}*-TLC1 TEL6R::FC70*
yT1612	yT1597 backcrossed with G49	S6	*MATa ura3-1 trp1-1::AFB2-TRP1 leu2-3.112 his3-11.15 can1-100 tlc1::HIS3MX6-*P_{Teto2}*-TLC1 TEL6R::FC70*
yT1690	CAN1-URA3 in trans FC70	6	*MATa ura3-1 trp1-1::AFB2-TRP1 leu2-3.112 his3-11.15 can1-100::CAN1-URA3 tlc1::HIS3MX6-*P_{Teto2}*-TLC1 TEL6R::FC70*
yT1694	CAN1-URA3 in trans	6	*MATa ura3-1 trp1-1::AFB2-TRP1 leu2-3.112 his3-11.15 can1-100::CAN1-URA3 tlc1::HIS3MX6-*P_{Teto2}*-TLC1*
yT1696	CAN1-URA3 in cis FC70 @12.5kb of last essential gene	S6	*MATa ura3-1 trp1-1::AFB2-TRP1 leu2-3.112 his3-11.15 can1-100 tlc1::HIS3MX6-*P_{Teto2}*-TLC1 CHR06:266663::CAN1-URA3 TEL6R::FC70*
yT1698	*CAN1-URA3* in *cis* @ 5 kb of last essential gene	S6-7	*MATa ura3-1 trp1-1::AFB2-TRP1 leu2-3.112 his3-11.15 can1-100 tlc1::HIS3MX6-*P_{Teto2}*-TLC1 CHR06:258991::CAN1-URA3 TEL6R::FC70*
yT1710	CAN1-URA3 in cis @12.5kb of last essential gene	S6	*MATa ura3-1 trp1-1::AFB2-TRP1 leu2-3.112 his3-11.15 can1-100 tlc1::HIS3MX6-*P_{Teto2}*-TLC1 CHR06:266663::CAN1-URA3*
yT1712	CAN1-URA3 in *cis* @ 5 kb of last essential gene	S6	*MATa ura3-1 trp1-1::AFB2-TRP1 leu2-3.112 his3-11.15 can1-100 tlc1::HIS3MX6-*P_{Teto2}*-TLC1 CHR06:258991::CAN1-URA3*
yT1759	CAN1-URA3 in *cis* @ 5 kb of last essential gene	6	*MATa ura3-1 trp1-1::AFB2-TRP1 leu2-3.112 his3-11.15 can1-Δ tlc1::HIS3MX6-*P_{Teto2}*-TLC1 CHR06:258991::CAN1-URA3*
yT1760	CAN1-URA3 in *cis* @ 5 kb of last essential gene	6. S7	*MATa ura3-1 trp1-1::AFB2-TRP1 leu2-3.112 his3-11.15 can1-Δ tlc1::HIS3MX6-*P_{Teto2}*-TLC1 CHR06:258991::CAN1-URA3 TEL6R::FC70*
yT1869	= yT1760 *exo1-Δ*	6	*MATa ura3-1 trp1-1::AFB2-TRP1 leu2-3.112 his3-11.15 can1-Δ tlc1::HIS3MX6-*P_{Teto2}*-TLC1 CHR06:258991::CAN1-URA3 TEL6R::FC70 exo1-Δ*
yT1870	= yT1760 *pol32-Δ*	6	*MATa ura3-1 trp1-1::AFB2-TRP1 leu2-3.112 his3-11.15 can1-Δ tlc1::HIS3MX6-*P_{Teto2}*-TLC1 CHR06:258991::CAN1-URA3 TEL6R::FC70 pol32-Δ*
yT1871	= yT1760 *rad1-Δ*	6	*MATa ura3-1 trp1-1::AFB2-TRP1 leu2-3.112 his3-11.15 can1-Δ tlc1::HIS3MX6-*P_{Teto2}*-TLC1 CHR06:258991::CAN1-URA3 TEL6R::FC70 rad1-Δ*
MCY813		5	*tlc1-Δ::HIS3/TLC1 leu2-3.112/LEU2-*P_{RNR3}*-TLC1 ura3::caURA3-*P_{RPL24A}*-FHA1-mCherry/ura3::caURA3-*P_{RPL24A}*-FHA1-mCherry hta2::HTA2-ECFP-KANMX6/hta2::HTA2-ECFP-KANMX6 can1-100/can1-100 his3-11.15/his3-11.15 trp1-1/trp1-1 RAD5/RAD5*

Supplementary Table 2: Bacteria strains used in this work

Name	Figure	Genotype
NEB® Stable Competent *E. coli* (High Efficiency)	3. S3	*F' proA+B+ lacIq Δ(lacZ)M15 zzf::Tn10 (TetR)/Δ(ara-leu) 7697 araD139 fhuA ΔlacX74 galK16 galE15 e14- Φ80dlacZΔM15 recA1 relA1 endA1 nupG rpsL (StrR) rph spoT1 Δ(mrr-hsdRMS-mcrBC)*
NEB® 10-beta Competent *E. coli* (High Efficiency)		*Δ(ara-leu) 7697 araD139 fhuA ΔlacX74 galK16 galE15 e14- ϕ80dlacZΔM15 recA1 relA1 endA1 nupG rpsL* (Str^R) *rph spoT1 Δ(mrr-hsdRMS-mcrBC)*

Supplementary Table 3: Plasmids used in this work

Name	Description	Source
pUC18	Empty backbone. pUC cloning vector	[1]
bRA77	Empty backbone. P_{Gal1}*-Cas9-AID-3myc*. gRNA can be cloned into BplI sites. Gift of J. Haber	[2]
bRA90	Encodes P_{PGK1}*-Cas9-3xFlag*. Gift of J. Haber	[2]
pRS305	Yeast integrative vector with a LEU2 marker.	[3]
pRS306	Yeast integrative vector with a URA3 marker.	[3]
pHis-AID*-9myc	C-terminal AID*-9myc degron cassette with HIS3MX marker	[4]
Ylp204-PADH1-AFB2	Expression of *PADH1-AFB2*	[4]
pT26	pURTel[5] derivative to modify *TEL6R*	[6]
pT63	Encodes P_{PGK1}*-Cas9-3xFlag*; sgRNA targets subtelomere 6R/*TEL6R* junction	This work
pT117	Encodes P_{Gal1}*-Cas9-AID-3myc* in a replicative plasmid	This work
pT122	Encodes P_{Gal1}*-Cas9-3xFlag* in an integrative plasmid	This work
pT126	Encodes P_{Gal1}*-Cas9-AID-3myc*; sgRNA targets FC	This work
pT182	For FC0 construction. contains fragment composed of subtelomere 6R followed by 5′-AAGCTTTTAGCATACGTGTGCGTACGCTCGAG**CCTGGATCGCAGTGGTGAGTAA***GAGCTC*GTGGGTGTGG…-3′[1]	This work
pT0177	For FC20 construction. contains fragment composed of subtelomere 6R followed by 5′-AAGCTTTTAGCATACGTGTGCGTACGCTCGAG**GTGGTGTGGGTGTGGTGTGTCCTGGGATCGCAGTGGTGAGTAA***GAGCTC*GTGGGTGTGG…-3′[1]	This work
pT176	For FC30 construction. contains fragment composed of subtelomere 6R followed by 5′-AAGCTTTTAGCATACGTGTGCGTACGCTCGAG**GTGGTGTGGGTGTGGTGTGTGGGTGTGGTGCCTGGGATCGCAGTGGTGAGTAA***GAGCTC*GTGGGTGTGG…-3′[1]	This work
pT175	For FC40 construction. contains fragment composed of subtelomere 6R followed by 5′-AAGCTTTTAGCATACGTGTGCGTACGCTCGAG**GTGGTGTGGGTGTGGTGTGTGGGTGTGGTGTGTGGGTGTGCCTGGGATCGCAGTGGTGAGTAA***GAGCTC*GTGGGTGTGG…-3′[1]	This work
pT174	For FC50 construction. contains fragment composed of subtelomere 6R followed by 5′-AAGCTTTTAGCATACGTGTGCGTACGCTCGAG**GTGGTGTGGGTGTGGTGTGTGGGTGTGGTGTGTGGGTGTGGTGTGGGTGTCCTGGGATCGCAGTGGTGAGTAA***GAGCTC*GTGGGTGTGG…-3′[1]	This work
pT187	For FC70 construction. contains fragment composed of subtelomere 6R followed by 5′-AAGCTTTTAGCATACGTGTGCGTACGCTCGAG**GTGGTGTGGGTGTGGTGTGTGGGTGTGGTGTGTGGGTGTGGTGTGGGTGTGTGGGTGTGTGGGTGTGGTCCTGGGATCGCAGTGGTGAGTAA***GAGCTC*GTGGGTGTGG…-3′[1]	This work
pT188	For FC100 construction. contains fragment composed of subtelomere 6R followed by 5′-AAGCTTTTAGCATACGTGTGCGTACGCTCGAG**GGTGGTGTGGGTGTGGTGTGTGGGTGTGGTGTGTGGGTGTGGTGTGGGTGTGTGGGTGTGTGGGTGTGGTGTGTGTGTGTGGGTGTGGGTGTGGGTGTGGCCTGGGATCGCAGTGGTGAGTAA***GAGCTC*GTGGGTGTGG…-3′[1]	This work
pIK2	P_{RNR3}*-TLC1* inserted into pRS305	This work
pT194	Encodes P_{PGK1}*-Cas9-Flag*. sgRNA targets *CAN1* locus	This work
pT196	*CAN1* locus and flanking region (ChrV 31457-34131) cloned into pRS306. Plasmid for CAN1-URA3 cassette amplification.	This work

[1] bold blue: telomeric repeats left at *FinalCut* telomere; **bold black: PAM and target sequence for Cas9 (reverse complement)**; *italics:* Sac *I site*; blue: terminal telomeric repeats.

Supplementary Table 4: Oligos used in this work

Name	5'-3' sequence
oT586	CAGGTATATCTATATGCTCTCATAGAATTATATTTGATATTGCTTTTTGGCGGATCCCCGGGTTAATTAA
oT587	TTTACTGGGCATTGATTTTTTAATTCTTGTCTTGAGGCATTTCGACGAGAGAATTCGAGCTCGTTTAAAC
oT633	CAGCTCGAAATAATATTTCACATTAACTAACAACCAGAAATAGGCTTTAGTTAACCGGATCCCCGGGTTAATTAA
oT634	GTAAGTTGACATTTGTATTATACATTACATCACAATTAGTAATGGAAAGTGTTTGGAATTCGAGCTCGTTTAAAC
oT0883	ACGTTTAGCTGAGTTTAACGGTG
oT1324	AACAAAGTTCGGGATTCAGCTCG
oT1450	GCCATATCAATATGCTAGTGGTTTT
oT1451	CACTAGCATATTGATATGGCGATCA
oT1550	TTACTCACCACTGCGATCCCGTTTT
oT1551	GGGATCGCAGTGGTGAGTAAGATCA
oT1572	ATGCACTAGTTGCACTAGGCG
oT1666	CTCCAGATTTATCAGCAATAAAC
oT1667	GTTTATTGCTGATAAATCTGGAG
oT1713	AGCTTTTAGCATACGTGTGCGTACGC
oT1714	TCGAGCGTACGCACACGTATGCTAAA
oT1741	GACTACAAAGACCATGACGGTGATTATCGTACGCTGCAGGTCGAC
oT1742	GTCGACCTGCAGCGTACGATAATCACCGTCATGGTCTTTGTAGTC
oT1749	GCCTGGATGACACGTAAATCAG
oT1750	CTGATTTACGTGTCATCCAGGC
oT1806	CTGTTTAGCTTGCCTCGTCCCTGATAATCTCTTCTCGAGTCATGT
oT1808	TCTCAATTGGGTGGTGATGAAGGGCGTACGCTGCAGGTCGAC
oT1829	CCTGTAGCATCGATAGCAGC
oT1830	GTTCGTTCCAATGTCAAGTTC
oT1831	CAAATGGTGAGACTGGAGAG
oT1832	GAAATCACGCCCTTTGTCCC
oT1833	TCACTCAAAGGCGGTAATAC
oT1834	CCTTTTGCTCACATGTTCTTT
oT1835	GAGTGCACCATATCGACTAC
oT1836	CCTTCTTGAACCATTTCCCA
oT1881	TTACTCACCACTGCGATCCC
oT1894	CGTGGATGATGTGGTCTCTACAG
oT1959	GGCTGGACTACTTTCTGGAATAGCG
oT1960	ACTGAGTTCGGATCACTACACACGG
oT1961	GATCCTAACGAGTGGATGCACAG
oT1962	GACCCAGTCCTCATTTCCATC
oT1963	GATGGTGGGGCAATTTCGAGAG
oT1964	CATGGCCATTCCTCAGGATCTTC
oT1965	CGATACCACGGCATTGATAAGC
oT1966	GGCACACTCGTACCATAAAACAG
oT2028	ATAGACCGAGATAGGGTTGAGTG
oT2029	GTGGACTCTTGTTCCAAACTGG
oT2032	CACACCCACACACCACACCCA
FW_RNR3pr+HindIII	ATCCTGAAGCTTAAGAGAAGGTAACAAGCACAT
REV_RNR3pr+BamHI	CAGGATGGATCCTTGTGTGGGAGTATTTGATTTATTG
FW_TLC1+BamHI	ATCCTGGATCCTGGTTTGAGAATAAAACTAGAGAGG
REV_TLC1+XbaI	CAGGATTCTAGAAAGAAGAAGCCATTTGGTGG
oT2057	ATGCAAGCTTTGAAATACCGGTTTT
oT2058	CGGTATTTCAAAGCTTGCATGATCA
oT2082	GGCGCGTCAGCGGGTGTTGGTATATCTTTAACAGATTCCAAAC
oT2083	GTTTGGAATCTGTTAAAGATATACCAACACCCGCTGACGCGCC
oT2084	GTAGTAAGCGCAACATACACCGGGTGTCGGGGCTGGC
oT2085	GCCAGCCCCGACACCCGGTGTATGTTGCGCTTACTAC
oT2098	TCCGGCACCAAGAATAGAGT
oT2126	GATTTTTTAGTTCGATTTCATTCAGTAGAGAAAGGCATATCAATATGACATGTATATCTTTAACAGATTCCAAAC
oT2127	TCTCTATTACGGTATTCTTTTTATTGCTAGCATTTAGCCGCGGAGTGAGATCAGTTTTGCTGGCCGCATCTTC
oT2130	GAAAATGCTGGTGTGAATGTGAATGACGATAGACGGACTGATGCACTTTTCCATTATATCTTTAACAGATTCCAAAC
oT2131	AGTCAAATTTTCAATCATGCTTCTCCCAATCTTGTAAGTAATGTTATCGTACAGTTTTGCTGGCCGCATCTTC
oT2132	TTTCTGTTGGTGCTGATATTGCNNNNNNNNNNACGTTTAGCTGAGTTTAACGGTG
oT2139	CCCAAAACAGTGCACCCAAG
oT2158	ACTTGCCTGTCGCTCTATCTTCGGGGGGGGGGG
oT2162	GAAGATAGAGCGACAGGCAAGTGCATGCAAGCTTGGCACTGG
oT2163	GCAATATCAGCACCAACAGAAAGAATTCGTAATCATGGTCATAGCTGT
oT2189	ATCGAACTGTGCGTGGAGAA
oT2190	TTCTTGGAGGCAGGGGATTG
oT2199	AGGCCACAGAACCGTATTCA
oT2276	TCAATCACTTACTGGCAAGTGCGTATAAATTAAACCTATTTCTTTATCATCATATTTACTTAAACCATTAAAGAATA TCTCGACCAGAATCTAACAGATATACATGTTCCGATAATGTCT
oT2387	CGGGATCCGGGGGGGGGGGGGGGGGG

oT2424	GAGCATTTGCTAAATGTGTAAAAATAATATTGCACTATCCTGTTGAAAATATCTTTCCAGCGGATCCCCGGGTTAATTAA
oT2425	CTATAGTTAATCGCATTTTATACTGATGTTTTAACAGGGTTCGTTAAATTAAACAATATTGAATTCGAGCTCGTTTAAAC

Supplementary Table 5: Composition of buffers used in Southern blots

	Hybond XL RPN303B GE Healthcare Amersham	Hybond N+RPN303B Amersham Hybond N+RPN303B
Hybridization buffer	Church buffer: 500 mM sodium phosphate pH 7.4. 1 mM EDTA; 7% SDS. 1% BSA	Modified Church and Gilbert buffer: phosphate buffer 0.5 M pH 7.2; SDS 7%; EDTA 10 mM
Low stringency wash	2× SSC and 0.1% SDS – 2 brief washes at RT	2x SSC. 0.1% SDS – one brief wash at RT; two washes 5 min 65°C
Medium stringency wash	1× SSC and 0.1% SDS – 1 wash of 15 min at 65°C	1x SSC. 0.1% SDS – two washes 10 min each 65°C
Medium stringency wash	0.5× SSC and 0.1% SDS – 1 wash of 15 min at 65°C	
High stringency wash	0.1× SSC and 0.1% SDS– 1-2 washes of 10 min at 65°C	0.1× SSC and 0.1% SDS – 4 washes 5 min at 65°C

Supplementary Table 6: Average ± s.e.m. (n=3) of fraction of uncut and cut bands in southern as in Fig.1d-e.

FinalCut	Hours in galactose	uncut	cut
FC20	0	1 ± 0	0 ± 0
	3	0.518 ± 0.169	0.091 ± 0.018
	6	0.216 ± 0.101	0.043 ± 0.017
	9	0.1 ± 0.03	0 ± 0
FC30	0	1 ± 0	0 ± 0
	3	0.575 ± 0.074	0.24 ± 0.02
	6	0.191 ± 0.022	0.167 ± 0.009
	9	0.102 ± 0.026	0.207 ± 0.04
FC40	0	1 ± 0	0 ± 0
	3	0.839 ± 0.076	0.15 ± 0.033
	6	0.369 ± 0.074	0.28 ± 0.054
	9	0.177 ± 0.051	0.32 ± 0.052
FC50	0	1 ± 0	0 ± 0
	3	0.427 ± 0.05	0.374 ± 0.058
	6	0.116 ± 0.031	0.513 ± 0.077
	9	0.078 ± 0.008	0.519 ± 0.033
FC70	0	0.991 ± 0.007	0.009 ± 0.007
	3	0.554 ± 0.064	0.459 ± 0.055
	6	0.21 ± 0.094	0.805 ± 0.037
	9	0.039 ± 0.021	0.967 ± 0.187

Supplementary Table 7: Fraction of cells in which the shortest telomere is the *FinalCut* telomere. computed from simulations of 50.000 lineages

FinalCut	Fraction of lineages in which the shortest telomere after the cut is *FinalCut*
FC20	1
FC30	1
FC40	1
FC50	1
FC70	0.999896773127980

Supplementary Table 8: Parameters fitted on the microfluidic data and used for the simulations

parameter	Value for the general model[7]	*FinalCut* (this work)
a_{nta}	0.02	0.02
b_{nta}	0.44	0.44
$a_{sen.A}$	0.19	0.17
$b_{sen.A}$	0.73	0.65
$a_{sen.B}$	0	0
$b_{sen.B}$	0.12	0.12

$l_{min.A}$	27	36
$l_{min.B}$	0	0
l_{trans}	0	0
l_0	40	40
l_1	58	58

Supplementary Table 9: Set of strains to study genomic instability in this work

position of *CAN1-URA3* cassette	*FC* status	*CAN1* @ chr 5L	Other mutations	strain name	Figure
In *trans* @ subtelomere 5L (@ *can1-100* locus)	*FC70*	*can1-100*		yT1690	6a.c.e
In *trans* @ subtelomere 5L (@ *can1-100* locus)	*noFC*	*can1-100*		yT1694	6a.c.e
In *cis* @12.5 kb from last essential gene on 6R arm	*FC70*	*can1-100*		yT1696	S6a.e.S7a
In *cis* @12.5 kb from last essential gene on 6R arm	*noFC*	*can1-100*		yT1710	S6e
In *cis* @ 5 kb from last essential gene on 6R arm	*FC70*	*can1-100*		yT1698	S6b.f.S7c-f
In *cis* @ 5 kb from last essential gene on 6R arm	*noFC*	*can1-100*		yT1712	S6f
In *cis* @ 5 kb from last essential gene on 6R arm	*FC70*	*can1-Δ*		yT1760	6b.d.f.7a-b.S7g-h
In *cis* @ 5 kb from last essential gene on 6R arm	*noFC*	*can1-Δ*		yT1759	6d
In *cis* @ 5 kb from last essential gene on 6R arm	*FC70*	*can1-Δ*	*pol32-Δ*	yT1870	6f
In *cis* @ 5 kb from last essential gene on 6R arm	*FC70*	*can1-Δ*	*exo1-Δ*	yT1869	6f
In *cis* @ 5 kb from last essential gene on 6R arm	*FC70*	*can1-Δ*	*rad1-Δ*	yT1871	6f

Supplementary Table 10: Results of fluctuation analyses applying canavanine selection

CAN1-URA3 cassette position	Time (h)	Relevant genotype	*CAN1* Mutation rate x 10^{-6} [95% Confidence interval]	n[1]	Mutation type distribution[a]: Point mutation	GCR	Others	n[2]
10 kb from the LEG[b] on arm 5L	0	*can1-100::CAN1-URA3 NoFC*	0.1 [0.08-0.12]	10	100	0	0	99
		can1-100::CAN1-URA3 FC70	0.08 [0.07-0.10]	16	100	0	0	68
	24	*can1-100::CAN1-URA3 NoFC*	0.08 [0.06-0.10]	10	100	0	0	48
		can1-100::CAN1-URA3 FC70	0.12 [0.09-0.16]	13	96.25±5.3	3.75±5.3	0	48
12.5 kb from the LEG[b] on arm 6R	0	*can1-100 NoFC*	0.33 [0.29-0.37]	9	54.5±6.4	45.5±6.4	0	99
		can1-100 FC70	0.66 [0.57-0.76]	5[c]	24.25±3.2	71.75±0.35	4±2.82	99
	24	*can1-100 NoFC*	0.37 [0.32-0.43]	14	36.75±1.8	63.25±1.77	0	92
		can1-100 FC70	8.26 [7.41-9.14]	5[c]	1±1.4	99±1.4	0	91
5 kb from the LEG[b] on arm 6R	0	*can1-100 NoFC*	0.18 [0.16-0.20]	15	76.7±9.9	20.9±13.34	2.4±4.22	129
		can1-100 FC70	0.41 [0.37-0.44]	15	30.7±15.3	65.3± 21.0	4±6.9	150
	24	*can1-100 NoFC*	0.16 [0.13-0.19]	15	61.7±4.5	38.3±4.47	0	128
		can1-100 FC70	6.96 [6.53-7.39]	15	2±2	97.3±1.15	0.7±1.15	200
	0	*can1-Δ NoFC*	0.12 [0.11-0.14]	19	100	0	0	239
		can1-Δ FC70	0.19 [0.16-0.22]	16	100	0	0	183
	24	*can1-Δ NoFC*	0.15 [0.12-0.18]	18	99.6±0.7	0.4±0.7	0	203
		can1-Δ FC70	0.68 [0.57-0.80]	16	35.9±14.6	64.1±14.6		313

n[1]: number of independent cultures
n[2]: number of Can[R] colonies analyzed
[a]Values are expressed in % ± SD over 2-3 independent experiments corresponding to n[1] cultures
[b]LEG: Last Essential Gene
[c]Experiment involving a single *FC70* transformant and experiment

Supplementary Table 11: Results of fluctuation analyses applying canavanine and 5-FOA double selection

CAN1-URA3 cassette position	Relevant Genotype	Media	*CAN1-URA3* Mutation rate x10^{-10}	[95% Confidence interval]	n[1]
10 kb from the LEG[b] on arm 5L (*TRANS*)	*can1-100::CAN1-URA3 FC70*	Raffinose	1.05	[0.24-2.25]	21
		Galactose	2.11	[0.69-4.06]	21
5 kb from the LEG[b] on arm 6R (*CIS*)	*can1-Δ FC70*	Raffinose	58.31	[41.31-77.39]	12
		Galactose	1044.99	[904.58-1192.76]	12
	can1-Δ pol32-Δ FC70	Raffinose	1.05	[0.19-2.37]	17
		Galactose	35.94	[21.65-52.77]	17

	can1-Δ exo1-Δ	*FC70*	Raffinose	35.89	[19.18-56.21]	10
			Galactose	933.92	[758.38-1122.63]	10
	can1-Δ rad1-Δ	*FC70*	Raffinose	136.69	[95.60-183.00]	10
			Galactose	1956.30	[1639.40-2293.50]	10

n[1]: number of independent cultures
[b]LEG: Last Essential Gene

Supplementary Table 12: Results of fluctuation analyses applying double selection – Ratios Gal/Raff

	TRANS	*CIS*			
	WT	*WT*	*pol32*	*exo1*	*rad1*
ratio Gal/Raff	2.01	17.92	34.23	26.02	14.31

Supplementary Table 13: Results of fluctuation analyses applying double selection – Ratios mutant/WT

	pol32		*exo1*		*rad1*	
	Raff	*Gal*	Raff	Gal	Raff	Gal
ratio mutant/WT	0.02	0.03	0.62	0.89	2.34	1.87

Supplementary Pipeline 1


```
<geneiousWorkflows>
  <XMLSerialisableRootElement name="TT_Clone&amp;Nanopore_Analysis1.1" author="teresatelott"
geneiousVersion="2023.2.1" uniqueId="d84ce14a-613a-490c-a277-fb89c20e7d5e" revisionNumber="15" description=""
bundledIconName="plugin">
    <workflowElement type="com.bioMATters.plugins.workflows.WorkflowElementForEach" />
    <workflowElement id="Geneious" exposeNoOptions="true" exposeAllOptions="false" suppressErrors="false"
showButtonForExposedGroup="false" groupNameForExposedOptions=""
type="com.bioMATters.plugins.workflows.DocumentOperationWorkflowElement">
      <Options>
        <option name="determineDirection">false</option>
        <option name="alignmentType">globalFreeEndGaps</option>
        <option name="nucleotideCostMATrix">70% similarity (IUB)</option>
        <option name="proteinCostMATrix">Blosum62</option>
        <option name="gapOpenPenalty">25.0</option>
        <option name="gapExtensionPenalty">5.0</option>
        <option name="refinementIterations">10</option>
        <option name="fastGuideTree">false</option>
        <option name="dontAlign">false</option>
      </Options>
      <optionToExpose optionName="determineDirection" label="" />
    </workflowElement>
    <workflowElement id="FilterOperation" exposeNoOptions="true" exposeAllOptions="false" suppressErrors="false"
showButtonForExposedGroup="false" groupNameForExposedOptions=""
type="com.bioMATters.plugins.workflows.DocumentOperationWorkflowElement">
      <Options>
        <option name="filterWhat">eachSequence</option>
        <option name="MATch">all</option>
        <multiOption name="filter">
          <value>
            <option name="field">percentage_similarity</option>
            <option name="condition">greater_than</option>
            <option name="value">95</option>
          </value>
        </multiOption>
      </Options>
      <optionToExpose optionName="filterWhat" label="" />
    </workflowElement>
```

```
  <workflowElement id="FilterOperation" exposeNoOptions="true" exposeAllOptions="false" suppressErrors="false"
showButtonForExposedGroup="false" groupNameForExposedOptions=""
type="com.bioMATters.plugins.workflows.DocumentOperationWorkflowElement">
    <Options>
      <option name="filterWhat">eachSequence</option>
      <option name="MATch">all</option>
      <multiOption name="filter">
        <value>
          <option name="field">percentage_identical</option>
          <option name="condition">greater_than</option>
          <option name="value">79.99999</option>
        </value>
      </multiOption>
    </Options>
    <optionToExpose optionName="filterWhat" label="" />
  </workflowElement>
  <workflowElement id="FilterOperation" exposeNoOptions="true" exposeAllOptions="false" suppressErrors="false"
showButtonForExposedGroup="false" groupNameForExposedOptions=""
type="com.bioMATters.plugins.workflows.DocumentOperationWorkflowElement">
    <Options>
      <option name="filterWhat">eachSequence</option>
      <option name="MATch">all</option>
      <multiOption name="filter">
        <value>
          <option name="field">meanCoverage</option>
          <option name="condition">greater_than</option>
          <option name="value">4.99999</option>
        </value>
      </multiOption>
    </Options>
    <optionToExpose optionName="filterWhat" label="" />
  </workflowElement>
  <workflowElement id="Generate_Consensus" exposeNoOptions="true" exposeAllOptions="false" suppressErrors="false"
showButtonForExposedGroup="false" groupNameForExposedOptions=""
type="com.bioMATters.plugins.workflows.DocumentOperationWorkflowElement">
    <Options>
      <option name="thresholdPercent">0</option>
      <option name="thresholdPercentNoQuality">65</option>
      <option name="noConsensusGaps">false</option>
      <option name="mapQuality">false</option>
      <option name="mapQualityMethod">mapSummed</option>
      <option name="noCoverageCharacterDeNovo">gap</option>
      <option name="noCoverageCharacterReference">gap</option>
      <option name="applyLowCoverageOrQualityCall">true</option>
      <option name="lowCoverageOrQualityCharacter">gap</option>
      <option name="coverageOrQuality">coverage</option>
      <option name="qualityThreshold">20</option>
      <option name="coverageThreshold">5</option>
      <option name="splitAroundQuestionMarks">false</option>
      <option name="noConsensusEndGaps">true</option>
      <option name="trimToReference">false</option>
      <option name="ignoreReadsMappedToMultipleLocations">false</option>
      <option name="removeGaps">true</option>
      <option name="appendText">true</option>
      <option name="textToAppend">consensus sequence</option>
      <option name="callWhenGapInBestStates" />
      <option name="callChroMATogramHeterozygotes">false</option>
      <option name="chroMATogramHeterozygotePercentage">50</option>
      <option name="howToStoreSequences">AskUser</option>
    </Options>
    <optionToExpose optionName="thresholdPercent" label="" />
  </workflowElement>
  <workflowElement type="com.bioMATters.plugins.workflows.WorkflowElementGroupSequences" />
```

```
    <workflowElement id="batchRename" exposeNoOptions="true" exposeAllOptions="false" suppressErrors="false"
showButtonForExposedGroup="false" groupNameForExposedOptions=""
type="com.bioMATters.plugins.workflows.DocumentOperationWorkflowElement">
      <Options>
        <option name="advancedCheckbox">false</option>
        <option name="customComponent1" />
        <childOption name="renameMethodChildOptions">
          <option name="selectedRenameMethod">renamableOption0</option>
          <childOption name="BatchRenameSubOptions0">
            <option name="attributeProcedure">nameProcedure</option>
            <option name="startBatchIndexOption">0</option>
            <option name="endBatchIndexInclusiveOption">2147483647</option>
            <option name="sequencesInSequenceListDocumentRenamableSequenceType">allSequencesInListOption</option>
          </childOption>
          <childOption name="BatchRenameSubOptions1">
            <option name="attributeProcedure">nameProcedure</option>
            <option name="followAndRenameReferences">true</option>
          </childOption>
          <childOption name="BatchRenameSubOptions2">
            <option name="selectedFieldComboOption">fieldOptioncache_name</option>
          </childOption>
        </childOption>
        <childOption name="basicSuperOptions">
          <option name="task">replaceWithRadioButton</option>
          <option name="r1">fieldOptiondocument_folder</option>
          <option name="rSep"> </option>
          <option name="r2">fieldOptioncache_name</option>
          <option name="rSep2" />
          <option name="r3">nothingSelectedOption</option>
          <option name="appendTextField" />
          <option name="appendPositionComboBox">endValue</option>
          <option name="removeHowMuchIntegerOption">1</option>
          <option name="removePositionComboBox">endValue</option>
        </childOption>
        <childOption name="advancedSuperOptions">
          <option name="replaceWhatTypeRadioOption">entireFieldOption</option>
          <option name="replaceWhatTextField" />
          <option name="ignoreCaseCheckbox">true</option>
          <option name="isRegexCheckbox">false</option>
          <option name="replaceWithTextField" />
          <option name="plusButton">Add Property...</option>
        </childOption>
      </Options>
      <optionToExpose optionName="advancedCheckbox" label="" />
    </workflowElement>
  </XMLSerialisableRootElement>
</geneiousWorkflows>
```

Supplementary Pipeline 2

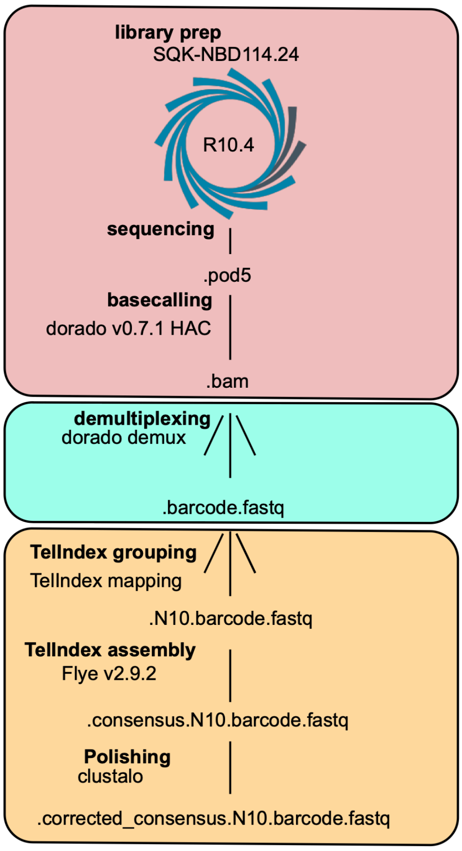

Analysis pipeline for high throughput individual telomere sequencing. DNA from TeloPCRs is prepared according to the SQK-SBD114.24 library protocol on a R10.4 Nanopore flowcell. Raw signal is then basecalled and demultiplexed. Individual telomeres are grouped according to their TelIndex tag. Grouped reads are assembled and consensus telomeres are multialigned in order to correct potential sequencing/assembly errors.

References

1 Norrander. J.. Kempe. T. & Messing. J. Construction of improved M13 vectors using oligodeoxynucleotide-directed mutagenesis. *Gene* **26**. 101–106 (1983). https://doi.org/10.1016/0378-1119(83)90040-9

2 Anand. R.. Beach. A.. Li. K. & Haber. J. Rad51-mediated double-strand break repair and mismatch correction of divergent substrates. *Nature* **544**. 377–380 (2017). https://doi.org/10.1038/nature22046

3 Sikorski. R. S. & Hieter. P. A system of shuttle vectors and yeast host strains designed for efficient manipulation of DNA in Saccharomyces cerevisiae. *Genetics* **122**. 19–27. (1989).

4 Morawska. M. & Ulrich. H. D. An expanded tool kit for the auxin-inducible degron system in budding yeast. *Yeast* **30**. 341–351 (2013). https://doi.org/10.1002/yea.2967

5 Gottschling. D. E.. Aparicio. O. M.. Billington. B. L. & Zakian. V. A. Position effect at S. cerevisiae telomeres: reversible repression of Pol II transcription. *Cell* **63**. 751–762 (1990). https://doi.org/10.1016/0092-8674(90)90141-z

6 Graf. M. *et al.* Telomere Length Determines TERRA and R-Loop Regulation through the Cell Cycle. *Cell* **170**. 72–85 e14 (2017). https://doi.org/10.1016/j.cell.2017.06.006

7 Rat. A.. Martinez Fernandez. V.. Doumic. M.. Teixeira. M. T. & Xu. Z. Mathematical model linking telomeres to senescence in Saccharomyces cerevisiae reveals cell lineage versus population dynamics. *Nat Commun* **16**. 1024 (2025). https://doi.org/10.1038/s41467-025-56196-z
